# Machine learning model for diagnosing salivary gland adenoid cystic carcinoma based on clinical and ultrasound features

**DOI:** 10.1186/s13244-025-01974-y

**Published:** 2025-05-08

**Authors:** Huan-Zhong Su, Zhi-Yong Li, Long-Cheng Hong, Yu-Hui Wu, Feng Zhang, Zuo-Bing Zhang, Xiao-Dong Zhang

**Affiliations:** 1 Department of Ultrasound, The First Affiliated Hospital of Xiamen University, School of Medicine, Xiamen University, Xiamen, China; 2 Department of Ultrasound, Fujian Medical University Union Hospital, Fuzhou, China

**Keywords:** Adenoid cystic carcinoma, Ultrasound, Machine learning, Rat tail sign, Polar vessel

## Abstract

**Objective:**

To develop and validate machine learning (ML) models for diagnosing salivary gland adenoid cystic carcinoma (ACC) in the salivary glands based on clinical and ultrasound features.

**Methods:**

A total of 365 patients with ACC or non-ACC of the salivary glands treated at two centers were enrolled in training cohort, internal and external validation cohorts. Synthetic minority oversampling technique was used to address the class imbalance. The least absolute shrinkage and selection operator (LASSO) regression identified optimal features, which were subsequently utilized to construct predictive models employing five ML algorithms. The performance of the models was evaluated across a comprehensive array of learning metrics, prominently the area under the receiver operating characteristic curve (AUC).

**Results:**

Through LASSO regression analysis, six key features—sex, pain symptoms, number, cystic areas, rat tail sign, and polar vessel—were identified and subsequently utilized to develop five ML models. Among these models, the support vector machine (SVM) model demonstrated superior performance, achieving the highest AUCs of 0.899 and 0.913, accuracy of 90.54% and 91.53%, and F1 scores of 0.774 and 0.783 in both the internal and external validation cohorts, respectively. Decision curve analysis further revealed that the SVM model offered enhanced clinical utility compared to the other models.

**Conclusions:**

The ML model based on clinical and US features provide an accurate and noninvasive method for distinguishing ACC from non-ACC.

**Critical relevance statement:**

This machine learning model, constructed based on clinical and ultrasound characteristics, serves as a valuable tool for the identification of salivary gland adenoid cystic carcinoma.

**Key Points:**

- Rat tail sign and polar vessel on US predict adenoid cystic carcinoma (ACC).
- Machine learning models based on clinical and US features can identify ACC.
- The support vector machine model performed robustly and accurately.

**Graphical Abstract:**

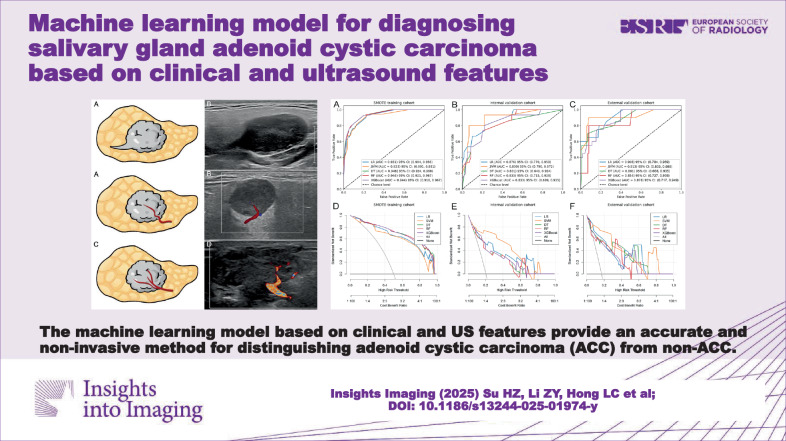

## Introduction

Adenoid cystic carcinoma (ACC) is an uncommon form of cancer, representing just 1% of all head and neck malignancies [1]. Predominantly affecting the salivary glands, it ranks as the second most prevalent malignant tumor in this region, following mucoepidermoid carcinoma [2]. It is important to recognize that ACC exhibits several malignant traits, such as locally invasive growth, local recurrence, and distant metastasis [3, 4]. The standard treatment for ACC involves surgical intervention, often accompanied by postoperative radiotherapy [5]. Notably, ACC has a high rate of distant metastasis, affecting nearly half of patients [6]. The most common sites for metastasis are the lungs, followed by bone and liver, with some cases emerging as long as 10 to 20 years post-diagnosis [7].

ACC presents a considerable diagnostic challenge and has garnered extensive research attention due to a lack of early clinical indicators, a low rate of early detection, a tendency for distant metastasis, and a poor long-term survival rate [8, 9]. Consequently, recognizing the distinctive behavior of ACC, an accurate preoperative imaging diagnosis is essential for devising the best treatment strategies. There is a pressing need to summarize the imaging characteristics of ACC. However, the rarity of this condition has resulted in most imaging studies being limited to isolated case reports or small case series [10, 11], which do not provide a comprehensive summary of its imaging features.

Ultrasound (US) is a non-invasive imaging modality that is extensively utilized for the detection of salivary gland lesions [12]. However, the body of knowledge regarding the use of US to identify ACC remains scarce. A profound comprehension of the US features of ACC is not only pivotal for distinguishing this specific malignancy from other salivary gland tumors, but it is also vital for optimizing clinical management.

Artificial intelligence methods employing machine learning (ML) algorithms could offer improved predictive insights for determining the best intervention strategies for individual patients [13]. ML’s ability to discern complex patterns from existing data has been shown to outperform conventional methods in forecasting medical outcomes [14]. Notably, ML predictive models have been extensively utilized in diagnosing, predicting treatment efficacy, and managing prognoses for head and neck tumors [15–18]. Considering that ACC of the salivary glands is uncommon, and its radiological features are not well elucidated. Thus, this retrospective study aimed to develop various predictive models for diagnosing salivary gland ACC based on ML algorithms by analyzing clinical and US characteristics of salivary gland tumors, and subsequently assess their effectiveness.

## Methods

### Patients

The research protocol was granted approval by the Ethics Committee of The First Affiliated Hospital of Xiamen University (protocol number 2024-107) and the Fujian Medical University Union Hospital (protocol number 2024WSJK028). Due to the retrospective design of the study, the committee exempted the need for obtaining informed consent from the participants. The study participants were enrolled from two different medical centers (center 1: The First Affiliated Hospital of Xiamen University; center 2: Fujian Medical University Union Hospital). Through a meticulous review of surgical and pathological documentation, this study conducted a comprehensive search of the electronic databases of center 1 and center 2. The search targeted patients who had been pathologically diagnosed with ACC of the salivary glands between January 2015 and July 2024. Additionally, this study searched the database for patients diagnosed with non-ACC of the salivary gland tumors from January 2022 to July 2024. Initially, 1150 patients were preliminarily identified for study inclusion. The exclusion criteria were: lack of US images, recurrent tumors, preoperative treatment history, unsatisfactory quality of US images, and incomplete US images of large lesions. Following these exclusion criteria, 785 patients were subsequently eliminated from the study. Ultimately, a total of 68 patients with ACC (mean age 50.82 ± 13.03 years; range 23–82 years; 32 male, 36 female) and 297 patients with non-ACC (mean age 47.70 ± 15.67 years; range 4–86 years; 190 male, 107 female) were included in the analysis. All patients underwent surgical resection, and the pathological findings were confirmed by experienced pathologists. Moreover, relevant clinical information for each patient was also extracted. To assess the potential impact of the different time frames for ACC patient inclusion on the study results, sensitivity analyses were conducted. The analyses compared key clinical and US characteristics between ACC patients diagnosed from January 2015 to December 2021 (*n* = 47) and those diagnosed from January 2022 to July 2024 (*n* = 21). No significant differences in key variables suggest that the time frame selection for ACC patients had minimal impact on the study outcomes.

**Fig. 1:**
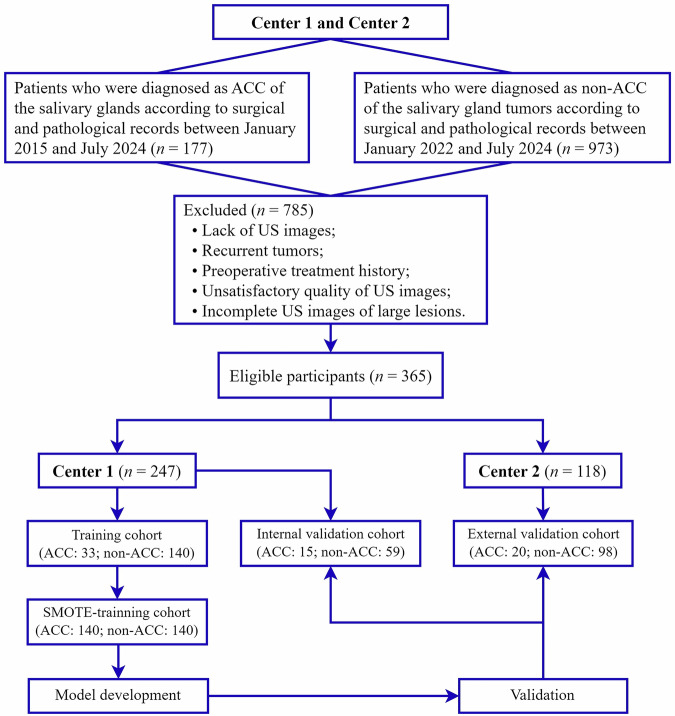
Analytic flowchart detailing cohort selection and exclusion criteria of the study. ACC, adenoid cystic carcinoma; US, ultrasound

In the next step, we employed random sampling to segment the research population from center 1 into a training cohort, consisting of 33 ACC cases and 140 non-ACC cases, and an internal validation cohort, comprising 15 ACC cases and 59 non-ACC cases, in a 7:3 proportion. Moreover, the research population from center 2 was designated as the external validation cohort, which included 20 ACC cases and 98 non-ACC cases. The workflow detailing the inclusion and exclusion criteria is presented in Fig. 1.

### US evaluation

All US examinations were performed by experienced radiologists with over 5 years of previous experience in performing neck US. The examinations were conducted utilizing advanced US equipment, including a Philips EPIQ7 (Philips Ultrasound), a GE Logiq E9 (GE Healthcare), and an Aixplorer ultrasound scanner (Supersonic Imagine). These devices were all equipped with high-resolution linear array probes, with frequency ranging from 6 to 15 MHz. Patients were positioned supine, with their necks fully exposed and heads tilted to the contralateral side, which allowed for the thorough scanning of various sections of both the parotid and submandibular gland regions. The clearest and most comprehensive US images were then obtained and recorded.

**Fig. 2:**
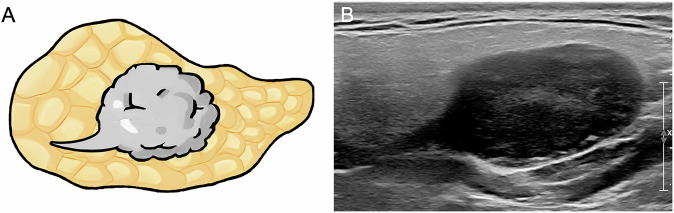
The rat tail sign is depicted as a thin tail-like hypoechoic pattern connected to one side of the tumor. **A** Schematic diagram. **B** Corresponding ultrasound image

**Fig. 3:**
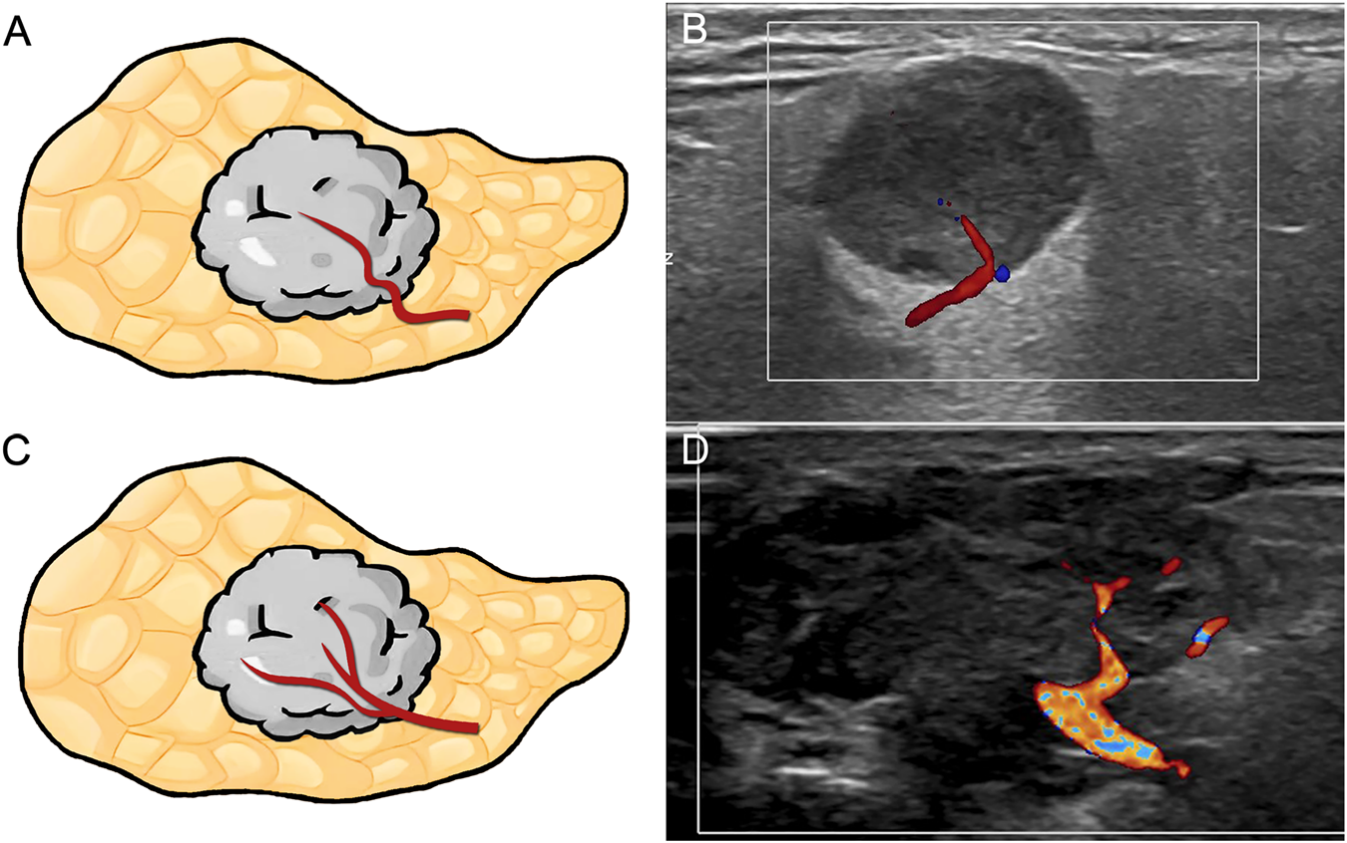
The polar vessel is depicted as a dominant vessel penetrating the tumor in color Doppler flow imaging. **A**, **B** Schematic diagram (**A**) and corresponding ultrasound image (**B**) illustrate the polar vessel as a dominant vessel without branches. **C**, **D** Schematic diagram (**C**) and corresponding ultrasound image (**D**) illustrate the polar vessel as a dominant vessel with branches

The US assessments meticulously documented various characteristics of the lesions, including size (recorded as the maximum length), number, border, shape, heterogeneity, echo, presence of cystic areas, calcification, degree of internal vascularity (grade 0, no visible tumor vessels; grade 1, consistent detection of 1 or 2 separate vessels; grade 2, consistent detection of 3 to 5 separate visible vessels; and grade 3, more than 5 separate visible vessels) [19], and presence or absence of rat tail sign and polar vessel. The rat tail sign was defined as a thin tail-like hypoechoic pattern connected to one side of the tumors (Fig. 2). The polar vessel was defined as a dominant vessel with or without branches penetrating the tumors in color Doppler flow imaging (Fig. 3). All US images were independently assessed by two senior radiologists, each with over 10 years of clinical experience, who were unaware of the histological pathology findings. In instances of disagreement, a third radiologist with equivalent experience was invited to arbitrate the final decision.

### Evaluation of interobserver reliability

Two radiologists with 5–10 years of clinical experience interpreted the US characteristics of all patients in a double-blind manner and conducted a Kappa consistency test on the results. The classification criteria for the consistency test results are as follows: 0.0 for poor agreement, 0.01–0.20 for slight agreement, 0.21–0.40 for fair agreement, 0.41–0.60 for moderate agreement, 0.61–0.80 for substantial agreement, and 0.81–0.99 for excellent agreement, with perfect agreement equating to 1.0.

### Oversampling

To address the challenge of dataset imbalance in our research, we employed the synthetic minority oversampling technique (SMOTE) [20] on the training cohort. This technique generates synthetic data that mirrors the structure of the original data from the minority class. Consequently, we produced a balanced dataset, which we refer to as the SMOTE-training cohort, enriched with SMOTE-generated samples.

### Feature selection, model development, and validation

We utilized the least absolute shrinkage and selection operator (LASSO) regression [21] on the SMOTE-training dataset (*n* = 280) to pinpoint the most effective clinical and US features. The penalty parameter was fine-tuned using a 10-fold cross-validation approach. The features selected by LASSO regression were then incorporated into five different ML algorithms: logistic regression (LR), support vector machine (SVM), decision tree (DT), random forest (RF), and extreme gradient boosting (XGBoost) to develop predictive models. The performance of these models was validated through an internal validation cohort and an external validation cohort. Hyperparameter settings for the various ML models are detailed in Supplementary Table 1. Model performance was assessed through a comprehensive set of metrics, including the area under the curve (AUC) from receiver operating characteristic (ROC) analysis, sensitivity, specificity, accuracy, positive predictive value (PPV), negative predictive value (NPV), F1 score, confusion matrix analysis, and decision curve analysis (DCA). To compare the AUCs across different models, we applied the DeLong test. Additionally, we conducted 10-fold cross-validation on the SMOTE-training cohort for the optimal model to further assess its performance.

### Statistical analysis

In our statistical analysis, we utilized a variety of software packages, including IBM SPSS Statistics (Version 22.0; https://www.ibm.com/spss), R (Version 4.4.1; https://www.r-project.org), and Python (Version 3.7.0; https://www.python.org/). We applied tests for normal distribution and homogeneity of variance to all quantitative variables. Descriptive statistics were presented as means ± standard deviations for variables with a normal distribution, and as medians and ranges for those that were not normally distributed. Qualitative variables were analyzed using the chi-square test and Fisher’s exact test. For quantitative variables, we employed independent samples *t*-tests for normally distributed data and Kruskal–Wallis H tests for data with a skewed distribution. The threshold for statistical significance was set at *p* < 0.05, considering two-tailed tests. For the R environment, all necessary packages can be downloaded https://cran.r-project.org/web/packages/.

## Results

### Baseline characteristics

The two radiologists achieved substantial to excellent agreement across all US features, with Kappa values ranging from 0.778 to 0.908 (Supplementary Table 2). Table 1 summarizes the baseline information for all patients in the study. There were no statistically significant differences in any characteristics among the three different cohorts. As shown in Table 2, significant differences between ACC and non-ACC were observed for sex, location, pain symptoms, border, shape, heterogeneity, cystic areas, rat tail sign, and polar vessel in the training cohort; for pain symptoms, border, cystic areas, rat tail sign, and polar vessel in the internal validation cohort; and for sex, location, cystic areas, rat tail sign, and polar vessel in the external validation cohort. No statistically significant differences were observed between ACC and non-ACC for other characteristics (*p* > 0.05). To further illustrate the US features of ACC, the US images of ACC are displayed in Supplementary Fig. 1.

**Table 1:** Baseline characteristics of patients

Characteristics	Training cohort (*n* = 173)	Internal validation cohort (*n* = 74)	External validation cohort (*n* = 118)	*p*-value
Group (ACC/non-ACC)	33/140	15/59	20/98	0.829
Age, years (mean ± SD)	49.54 ± 14.73	46.66 ± 15.72	47.05 ± 15.68	0.252
Sex (male/female)	112/61	41/33	69/49	0.317
Location (PG/SMG)	132/41	52/22	93/25	0.398
Smoking history (absence/presence)	101/72	50/24	71/47	0.393
Pain symptoms (absence/presence)	154/19	68/6	100/18	0.296
Size, cm (mean ± SD)	2.81 ± 1.03	2.61 ± 0.98	2.58 ± 1.22	0.154
Number (one/multiple)	158/15	66/8	104/14	0.660
Border (clear/unclear)	146/27	66/8	106/12	0.333
Shape (regular/irregular)	100/73	34/40	67/51	0.207
Heterogeneity (homogeneous/heterogeneous)	94/79	39/35	64/54	0.970
Echo (hypoechoic/isoechoic or hyperechoic)	166/7	69/5	116/2	0.182
Cystic areas (absence/presence)	87/86	39/35	71/47	0.245
Calcification (absence/presence)	159/14	70/4	110/8	0.742
Vascularity (grade 0–1/grade 2–3)	60/113	34/40	49/69	0.206
Rat tail sign (absence/presence)	149/24	65/9	105/13	0.765
Polar vessel (absence/presence)	141/32	61/13	104/14	0.299

*ACC* adenoid cystic carcinoma, *PG* parotid gland, *SMG* submandibular gland

**Table 2:** Comparison of patients’ clinical and US characteristics between ACC and non-ACC in the training, internal validation, and external validation cohorts

Characteristics	Training cohort (*n* = 173)			Internal validation cohort (*n* = 74)			External validation cohort (*n* = 118)		
	ACC (*n* = 33)	Non-ACC (*n* = 140)	*p*-value	ACC (*n* = 15)	Non-ACC (*n* = 59)	*p*-value	ACC (*n* = 20)	Non-ACC (*n* = 98)	*p*-value
Age, years (mean ± SD)	48.70 ± 13.83	49.74 ± 14.97	0.715	52.61 ± 13.17	45.32 ± 15.34	0.069	48.80 ± 15.62	46.69 ± 15.75	0.586
Sex (male/female)	16/17	96/44	0.023*	8/7	33/26	0.857	8/12	61/37	< 0.001*
Location (PG/SMG)	20/13	112/28	0.018*	8/7	44/15	0.197	11/9	82/16	0.010*
Smoking history (absence/presence)	18/15	83/57	0.619	9/6	41/18	0.695	15/5	56/42	0.159
Pain symptoms (absence/presence)	24/9	130/10	0.003*	11/4	57/2	0.016*	16/4	84/14	0.759
Size, cm (mean ± SD)	2.74 ± 1.26	2.82 ± 0.98	0.714	3.03 ± 1.07	2.50 ± 0.93	0.060	2.25 ± 1.12	2.64 ± 1.23	0.184
Number (one/multiple)	32/1	126/14	0.349	14/1	52/7	0.910	20/0	84/14	0.155
Border (clear/unclear)	20/13	126/14	< 0.001*	9/6	57/2	< 0.001*	17/3	89/9	0.705
Shape (regular/irregular)	12/21	88/52	0.006*	4/11	30/29	0.093	8/12	59/39	0.096
Heterogeneity (homogeneous/heterogeneous)	11/22	83/57	0.007*	7/8	32/27	0.600	11/9	53/45	0.940
Echo (hypoechoic/isoechoic or hyperechoic)	32/1	134/6	1.000	14/1	55/4	1.000	20/0	96/2	1.000
Cystic areas (absence/presence)	27/6	60/8	< 0.001*	12/3	27/32	0.025*	17/3	54/44	0.013*
Calcification (absence/presence)	28/5	131/9	0.194	14/1	56/3	1.000	18/2	92/6	0.888
Vascularity (grade 0–1/grade 2–3)	13/20	47/93	0.527	10/5	24/35	0.071	12/8	37/61	0.066
Rat tail sign (absence/presence)	20/13	129/11	< 0.001*	9/6	56/3	0.001*	9/11	96/2	< 0.001*
Polar vessel (absence/presence)	11/22	130/10	< 0.001*	6/9	55/4	< 0.001*	9/11	95/3	< 0.001*

*US* ultrasound, *ACC* adenoid cystic carcinoma, *PG* parotid gland, *SMG* submandibular gland

* Statistically significant at *p* < 0.05

### Histological types of patients with salivary gland tumors

A detailed presentation of the histological types of patients with salivary gland tumors and the presence of the rat tail sign and polar vessel in each pathological subtype is provided in Table 3. Of 365 patients with salivary gland tumors, there were 68 ACC and 297 non-ACC (120 pleomorphic adenoma, 78 Warthin tumor, 19 basal cell adenoma, 15 mucoepidermoid carcinoma, 7 acinar cell carcinoma, 6 salivary duct carcinoma, 6 carcinoma ex pleomorphic adenoma, 10 lymphoma, 10 salivary gland metastases, and 26 others). Statistically significant differences were noted in the US characteristics of the rat tail sign and polar vessel between ACC and the diverse non-ACC pathological types (all *p* < 0.001).

**Table 3:** The presence of rat tail sign and polar vessel in different histologic types of patients with salivary gland tumors

Types	Patients (*n* = 365)	Rat tail sign (*n* = 46)	Polar vessel (*n* = 59)	*p*-value^a^	*p*-value^b^
Adenoid cystic carcinoma	68	30 (44.1%)	42 (61.8%)		
Nonadenoid cystic carcinoma					
Pleomorphic adenoma	120	6 (5.0%)	7 (5.8%)	< 0.001*	< 0.001*
Warthin tumor	78	5 (6.4%)	6 (7.7%)	< 0.001*	< 0.001*
Basal cell adenoma	19	1 (5.3%)	1 (5.3%)	< 0.001*	< 0.001*
Mucoepidermoid carcinoma	15	1 (6.7%)	0 (0%)	< 0.001*	< 0.001*
Acinar cell carcinoma	7	0 (0%)	1 (14.3%)	< 0.001*	< 0.001*
Salivary duct carcinoma	6	0 (0%)	0 (0%)	< 0.001*	< 0.001*
Carcinoma ex pleomorphic adenoma	6	0 (0%)	0 (0%)	< 0.001*	< 0.001*
Lymphoma	10	1 (10.0%)	0 (0%)	< 0.001*	< 0.001*
Salivary gland metastases	10	1 (10.0%)	1 (10.0%)	< 0.001*	< 0.001*
Others	26	1 (3.9%)	1 (3.9%)	< 0.001*	< 0.001*

Data are presented as numbers (percentages)

* Statistically significant at *p* < 0.05

^a^ *p*-value for the statistical test comparing the presence of rat tail sign with adenoid cystic carcinoma

^b^ *p*-value for the statistical test comparing the presence of polar vessel with adenoid cystic carcinoma

### Clinical and US characteristics in the SMOTE-training cohort

Table 4 presents a comprehensive overview of the clinical and US characteristics of patients in the SMOTE-training cohort. Significant differences between the groups were observed in terms of sex, lesion location, pain symptoms, number, border, shape, heterogeneity, cystic areas, rat tail sign, and polar vessel (*p* < 0.05). No statistically significant differences were observed between the groups for other characteristics (*p* > 0.05).

**Table 4:** Comparison of patients’ clinical and US characteristics between ACC and non-ACC in the SMOTE-training cohort

Characteristics	ACC (*n* = 140)	Non-ACC (*n* = 140)	*p*-value
Age, years (mean ± SD)	48.56 ± 13.07	49.74 ± 14.97	0.484
Sex (male/female)	65/75	97/43	< 0.001*
Location (PG/SMG)	87/53	112/28	< 0.001*
Smoking history (absence/presence)	85/55	83/57	0.807
Pain symptoms (absence/presence)	112/28	130/10	0.002*
Size, cm (mean ± SD)	2.87 ± 1.13	2.82 ± 0.98	0.690
Number (one/multiple)	137/3	126/14	0.006*
Border (clear/unclear)	107/33	126/14	0.002*
Shape (regular/irregular)	71/69	88/52	0.040*
Heterogeneity (homogeneous/heterogeneous)	60/80	83/57	0.006*
Echo (hypoechoic/isoechoic or hyperechoic)	136/4	134/6	0.520
Cystic areas (absence/presence)	120/20	60/80	< 0.001*
Calcification (absence/presence)	125/15	131/9	0.200
Vascularity (grade 0–1/grade 2–3)	43/97	47/93	0.609
Rat tail sign (absence/presence)	104/36	129/11	< 0.001*
Polar vessel (absence/presence)	62/78	130/10	< 0.001*

*US* ultrasound, *ACC* adenoid cystic carcinoma, *SMOTE* synthetic minority oversampling technique, *PG* parotid gland, *SMG* submandibular

* Statistically significant at *p* < 0.05

### Feature selection and model development

**Fig. 4:**
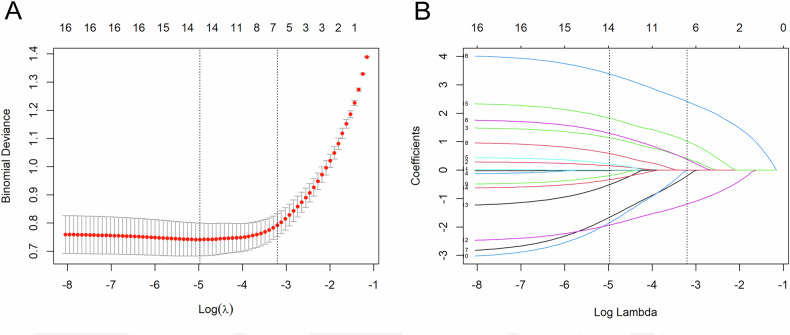
Feature selection employing LASSO Regression. **A** The LASSO tuning parameter (λ) was determined using a 10-fold cross-validation approach in conjunction with the 1 standard error rule. The optimal λ values are denoted by the dotted vertical lines, with the specific selection of λ = 0.041, which corresponds to a log(λ) of −3.207, indicated on the right. **B** The LASSO coefficient profiles were constructed for 16 clinical and ultrasound features. These profiles were plotted against the selected log(λ) value, obtained from 10-fold cross-validation, culminating in the identification of six features with substantial non-zero coefficients for final inclusion. LASSO, least absolute shrinkage and selection operator

Through LASSO regression analysis in the SMOTE-training cohort, six features were identified, comprising two clinical characteristics (sex, pain symptoms) and four US features (number, cystic areas, rat tail sign, and polar vessel). The optimal regularization parameter λ was determined to be 0.041, with a log(λ) value of −3.207 under the criterion of 1 standard error (Fig. 4). Subsequently, various ML-based predictive models were constructed utilizing the selected six features.

### The discrimination performance of the different model

**Fig. 5:**
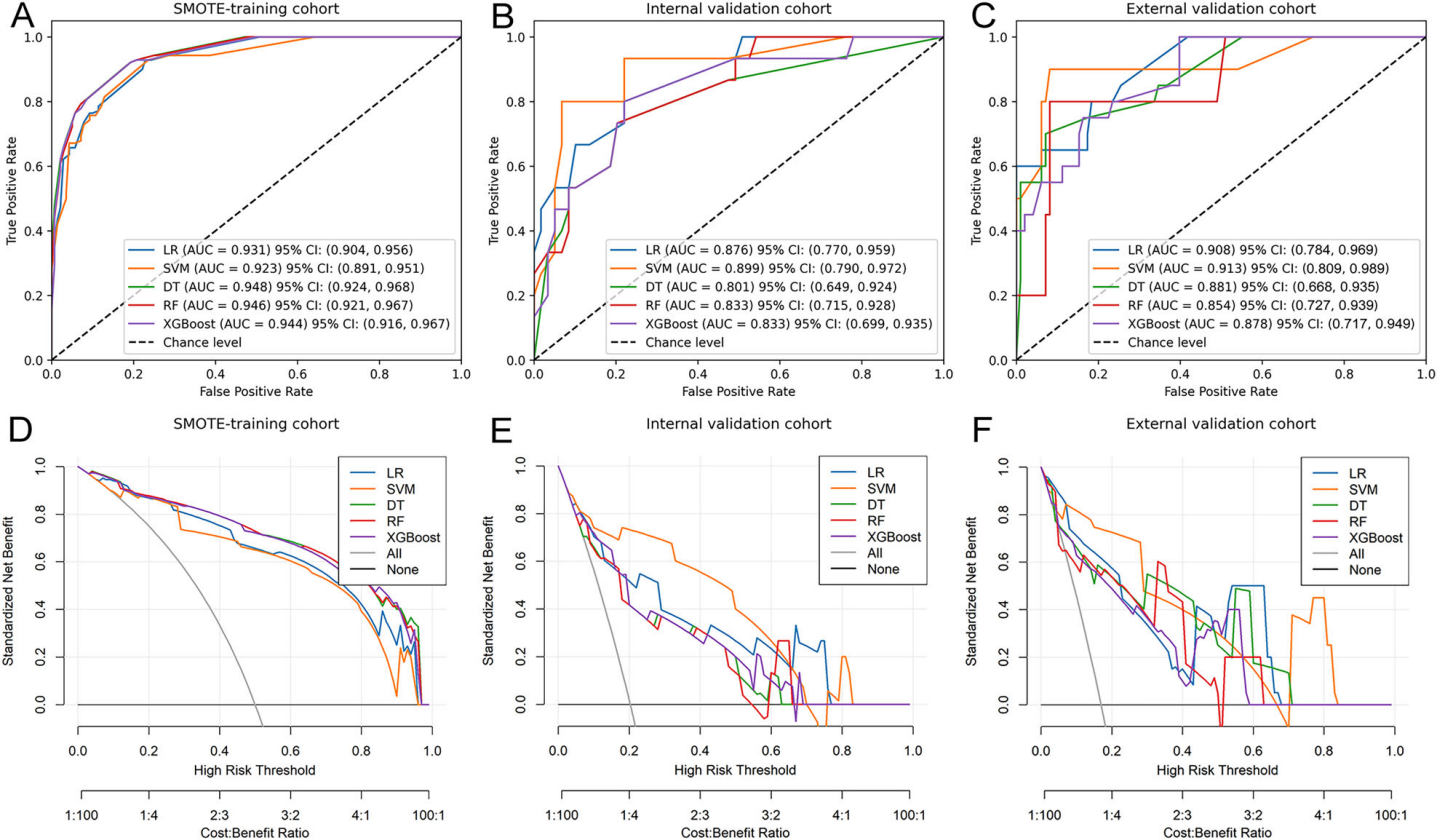
ROC curves and DCA of the different predictive models. ROC curves of the different models in the SMOTE-training (**A**), internal validation (**B**) and external validation (**C**) cohorts. DCA of the different predictive models in the SMOTE-training (**D**), internal validation (**E**) and external validation (**F**) cohorts. ROC, receiver operating characteristic curve; DCA, decision curve analysis; SMOTE, synthetic minority oversampling technique; AUC, area under curve; LR, logistic regression; SVM, support vector machine; DT, decision tree; RF, random forest; XGBoost, extreme gradient boosting; CI, confidence interval

The ROC curves for the models in the SMOTE-training cohort, internal validation cohort, and external validation cohort are depicted in Fig. 5A–C. Table 5 presents the performance of various predictive models in both the internal and external validation cohorts, while Supplementary Figs. 2 and 3 display the corresponding confusion matrices for different predictive models in the internal and external validation cohorts. The current analysis reveals that the AUC ranges for the five ML models in the internal and external validation cohorts are 0.801–0.899 and 0.854–0.913, respectively. The SVM model performed the best in both the internal and external validation cohorts, with pairwise comparisons of AUCs between different predictive models shown in Supplementary Figs. 1F and 2F. The SVM model, using 10-fold cross-validation in the SMOTE-training cohort, achieved average AUCs of 0.893 ± 0.027 (Supplementary Fig. 4). Furthermore, we assessed the clinical value of these models; the DCA indicates (Fig. 5D–F) that for the discrimination between ACC and non-ACC, the SVM model provided a higher overall net benefit compared to other models across most reasonable threshold probability ranges in both the internal and external validation cohorts.

**Table 5:** Diagnostic performance of different ML models in both the internal and external validation cohorts for distinguishing ACC from non-ACC

	AUC (95% CI)	Sen (%)	Spe (%)	Acc(%)	PPV (%)	NPV (%)	F1 score
Internal validation cohort (*n* = 74)							
LR	0.876 (0.770–0.959)	80 (12/15)	77.97 (46/59)	78.38 (58/74)	48 (12/25)	93.88 (46/49)	0.6
SVM	0.899 (0.790–0.972)	80 (12/15)	93.22 (55/59)	90.54 (67/74)	75 (12/16)	94.83 (55/58)	0.774
DT	0.801 (0.649–0.924)	73.33 (11/15)	79.66 (47/59)	78.38 (58/74)	47.83 (11/23)	92.16 (47/51)	0.579
RF	0.833 (0.715–0.928)	73.33 (11/15)	79.66 (47/59)	78.38 (58/74)	47.83 (11/23)	92.16 (47/51)	0.579
XGBoost	0.833 (0.699–0.935)	80 (12/15)	77.97 (46/59)	78.38 (58/74)	48 (12/25)	93.88 (46/49)	0.6
External validation cohort (*n* = 118)							
LR	0.908 (0.784–0.969)	80 (16/20)	81.63 (80/98)	81.36 (96/118)	47.06 (16/34)	95.24 (80/84)	0.593
SVM	0.913 (0.809–0.989)	90 (18/20)	91.84 (90/98)	91.53 (108/118)	69.23 (18/26)	97.83 (90/92)	0.783
DT	0.881 (0.668–0.935)	70 (14/20)	92.86 (91/98)	88.98 (105/118)	66.67 (14/21)	93.81 (91/97)	0.683
RF	0.854 (0.727–0.939)	80 (16/20)	91.84 (90/98)	89.83 (106/118)	66.67 (16/24)	95.74 (90/94)	0.727
XGBoost	0.878 (0.717–0.949)	100 (20/20)	60.2 (59/98)	66.95 (79/118)	33.9 (20/59)	100 (59/59)	0.506

Data in the parentheses are raw data

*ML* machine learning, *ACC* adenoid cystic carcinoma, *LR* logistic regression, *SVM* support vector machine, *DT* decision tree, *AUC* area under receiver operating characteristics, *CI* confidence interval, *Sen* sensitivity, *Spe* specificity, *Acc* accuracy, *PPV* positive predictive value, *NPV* negative predictive value, *RF* random forest, *XGBoost* extreme gradient boosting

## Discussion

ACC accounts for 25% of malignant tumors in the major salivary glands and 50% in the minor salivary glands [22]. Despite its generally indolent growth pattern, ACC has an unfavorable prognosis, attributable to its propensity for diffuse invasion, frequent local recurrence, and a high rate of distant metastasis [3, 4]. As a result, an accurate preoperative imaging diagnosis is needed to determine the nature of the tumor. Nevertheless, there has been limited exploration of the US characteristics of ACC and the distinctions between ACC and other salivary gland tumors. Therefore, this study seeks to provide valuable insights to the existing body of knowledge in this field. In our study, we developed a predictive model for preoperative classification between ACC and non-ACC. By employing the LASSO regression algorithm for feature selection, we identified six key features and utilized a variety of ML algorithms to develop and validate the predictive model. To our knowledge, this study represents the largest sample size to date in investigating the clinical and US characteristics of ACC in the salivary glands. It is also the first study to employ ML algorithms to construct predictive models that distinguish ACC from non-ACC based on clinical and US features. In both internal and external validation cohorts, the SVM model demonstrated the highest predictive accuracy, with AUC reaching 0.899 and 0.913, respectively. Furthermore, the results from DCA indicated that the SVM model has a more significant advantage in clinical application compared to other models.

Regarding epidemiology and clinical characteristics, ACC generally arises in the third to ninth decades, with an average of the fifth decade, and exhibits a slight female predominance [23, 24]. In the present study, the ratio of women:men was 1.1 (36/32) for the patients with ACC, with an average age of 50.82 years, which is consistent with previous epidemiological studies. Previous research has indicated that ACC in the major salivary glands is more frequently located in the submandibular gland, and submandibular gland ACC carries a worse prognosis than other gland subsites [25]. In our study, a higher proportion of ACC cases were located in the submandibular gland when compared to non-ACC cases; however, the proportion of cases in the parotid gland within the ACC group was still higher than in the submandibular gland, possibly due to an uneven distribution of the sample. In addition, pain symptoms have been reported in association with ACC in previous studies [26], and our study found similar results, with a higher incidence of facial pain symptoms in ACC patients compared to non-ACC patients. Our study did not, however, show significant differences in age and smoking history between ACC and non-ACC patients.

In our study, regarding US features of ACC alone, most lesions may exhibit diverse manifestations typical of salivary gland malignancy [12], such as unclear borders, irregular shapes, solid hypoechoic patterns, as well as heterogeneous internal echoes. In comparison to non-ACC, ACC is more likely to present with solitary lesions, unclear borders, irregular shapes, heterogeneous internal echoes, absence of cystic areas, and the presence of rat tail sign and polar vessel. The analysis of interobserver variability for these US characteristics revealed a high degree of substantial and excellent agreement. Among the 68 cases of ACC, 30 showed the rat tail sign (44.1%, 30/68), and 42 exhibited polar vessel (61.8%, 42/68). When comparing ACC to other histological types of salivary gland tumors, the presence of rat tail sign and polar vessel was significantly different, underscoring their diagnostic value. Conversely, characteristics such as lesion size, echo, calcification and internal vascularity cannot be relied upon for differential diagnosis.

ACC is known for its propensity for perineural and perivascular invasion, which is reflected in the high incidence of local recurrence and the tendency for late-onset distant metastasis observed clinically [27, 28]. Perineural invasion (PNI), occurring asymptomatically in 40% of ACC patients, necessitates high-resolution imaging for preoperative assessment of nerve invasion pathways [29]. Magnetic resonance imaging has proven particularly effective in detecting PNI, with key findings including fat replacement around neural foramina, enlargement of neural foramina, diffuse or nodular nerve thickening, and nerve enhancement [30]. Hanna et al found that magnetic resonance imaging detects PNI more sensitively than computed tomography, with respective sensitivities of 100% and 88%, and recommended using pre- and post-enhanced T1-weighted images and fat-saturated contrast-enhanced T1-weighted images for visualizing PNI in head and neck tumors [29]. PNI of ACC is a well-established prognostic factor strongly associated with increased risk of local recurrence [31]. Our imaging observations of the rat tail sign may represent a distinctive feature indicative of local invasion and PNI in ACC. This finding aligns with the longitudinal tail sign identified by Wang et al [32] as a characteristic CT feature of tracheal ACC, suggesting a potential common imaging manifestation across different anatomical sites. The consistency of these findings across studies further underscores the aggressive behavior and metastatic patterns of ACC. Additionally, the incidence of perivascular invasion in ACC is notably high, reaching up to 15%, and is associated with a high rate of pulmonary metastasis [33]. The identification of polar vessels in our study corroborates previous observations [10, 34], reinforcing the diagnostic significance of perivascular spread patterns in salivary gland ACC. These unique imaging features, including the rat tail sign and the polar vessel appearance, provide valuable diagnostic clues that may enhance the preoperative assessment and management of ACC.

In this study, we employed a feature selection process using LASSO regression analysis to further identify six key features that have the most significant impact in discriminating between ACC and non-ACC. These include two clinical characteristics (sex, pain symptoms) and four US features (number, cystic areas, rat tail sign, and polar vessel). By decreasing the number of features, we not only streamlined the model but also bolstered its interpretability and broader applicability. The model was further optimized through the determination of the ideal regularization parameter λ via cross-validation, which helped to reduce overfitting and enhance predictive precision [21].

In terms of predictive performance, the ML models demonstrated AUC values ranging from 0.801 to 0.899 in the internal validation cohort and from 0.854 to 0.913 in the external validation cohort, indicating varying effectiveness in distinguishing between ACC and non-ACC. Our results indicate that the SVM is a powerful model with strong generalization capabilities, achieving the highest AUC (0.899 and 0.913, respectively), accuracy (90.54% and 91.53%, respectively), and F1 score (0.774 and 0.783, respectively) in both internal and external validation cohorts. The SVM algorithm excels at separating data by projecting it into a higher-dimensional space and is capable of managing non-linear complexities through the selection of suitable kernel functions [35]. In building the model, SVM upholds the principle of minimizing structural risk, with the goal of achieving the lowest possible generalization error [36]. DCA substantiated the clinical relevance of the SVM model, showing that it offers greater overall net benefit across a wide array of threshold probabilities in both internal and external validation sets. This indicates that the SVM model is more apt to accurately identify ACC, thus enabling more knowledgeable surgical planning.

Despite the significance of our findings, it is important to recognize several limitations inherent to our study. First, the retrospective nature of the study may have introduced potential selection bias. Second, the sample size was relatively small. Although the number of patients in this retrospective analysis exceeded that of many prior studies, the rarity of ACC led to a limited sample size. Consequently, future research should aim to include a larger and more extensive cohort to more definitively characterize the imaging features of ACC and provide higher-level evidence for clinical application. Additionally, the scans were conducted by sonographers with varying levels of experience, and some failed to document the rat tail sign and polar vessel due to a lack of awareness. Lastly, our investigation was confined to conventional US characteristics, and unfortunately, data from advanced US techniques such as sonoelastography or contrast-enhanced US were not fully available. Future studies should endeavor to incorporate multimodal US features to accurately distinguish ACC from non-ACC.

In conclusion, our research has confirmed that ML models based on clinical and US features are indeed feasible and promising for distinguishing ACC from non-ACC, with the SVM model showing particular potential in enhancing diagnostic accuracy. Recent advances in radiomics and deep learning have further demonstrated their efficacy in salivary gland tumor diagnosis [16, 37–39], highlighting their ability to improve tumor characterization and automate feature extraction. Future studies should focus on optimizing these models, integrating multimodal datasets, and validating their clinical utility to maximize patient benefits, while exploring AI’s broader applications in ACC diagnosis and management.

## Supplementary information

ELECTRONIC SUPPLEMENTARY MATERIAL

## Data Availability

The data generated during and/or analyzed during the current study are available from the corresponding author on reasonable request.

## Abbreviations

ACC: Adenoid cystic carcinoma
AUC: Area under curve
DCA: Decision curve analysis
DT: Decision tree
LASSO: Least absolute shrinkage and selection operator
LR: Logistic regression
ML: Machine learning
NPV: Negative predictive value
OR: Odds ratio
PG: Parotid gland
PNI: Perineural invasion
PPV: Positive predictive value
ROC: Receiver operating characteristic
SMG: Submandibular gland
SVM: Support vector machine
US: Ultrasound
XGBoost: Extreme gradient boosting
