# Supplementary material for: Machine learning model for diagnosing salivary gland adenoid cystic carcinoma based on clinical and ultrasound features
Source: Insights Imaging. 2025 May 8;16:96. doi: 10.1186/s13244-025-01974-y (PMC12061827; doi:10.1186/s13244-025-01974-y)
Supplement: Supplementary file 1 — ELECTRONIC SUPPLEMENTARY MATERIAL [file 13244_2025_1974_MOESM1_ESM.pdf]

Machine learning model for diagnosing salivary gland adenoid cystic carcinoma based on clinical and ultrasound features

ELECTRONIC SUPPLEMENTARY MATERIAL

Supplementary Table 1 Hyperparameter settings of the different prediction models.

	Hyperparameter settings
**LR**	'C': 10, 'penalty': 'l1'
**SVM**	'C': 0.1, 'gamma': 'scale', 'kernel': 'linear'
**DT**	'criterion': 'gini', 'max_depth': None, 'min_samples_leaf': 1, 'min_samples_split': 2
**RF**	'bootstrap': False, 'max_depth': 20, 'min_samples_leaf': 1, 'min_samples_split': 10, 'n_estimators': 300
**XGBoost**	'colsample_bytree': 0.9, 'learning_rate': 0.2, 'max_depth': 3, 'n_estimators': 100, 'subsample': 0.9

Note: LR, logistic regression; SVM, support vector machine; DT, decision tree; RF, random forest; XGBoost, extreme gradient boosting.

Supplementary Table 2 Interobserver variability of US features.

Characteristics	Kappa	95% CI	P value
Border	0.811	0.732 - 0.889	< 0.001*
Shape	0.841	0.787 - 0.896	< 0.001*
Heterogeneity	0.794	0.733 - 0.855	< 0.001*
Echo	0.793	0.613 - 0.973	< 0.001*
Cystic areas	0.905	0.862 - 0.948	< 0.001*
Calcification	0.908	0.828 - 0.988	< 0.001*
Vascularity	0.778	0.713 - 0.843	< 0.001*
Rat tail sign	0.871	0.782 - 0.959	< 0.001*
Polar vessel	0.860	0.778 - 0.942	< 0.001*

Note: US, ultrasound; CI, confidence interva. *Statistically significant at $P < 0.05$.

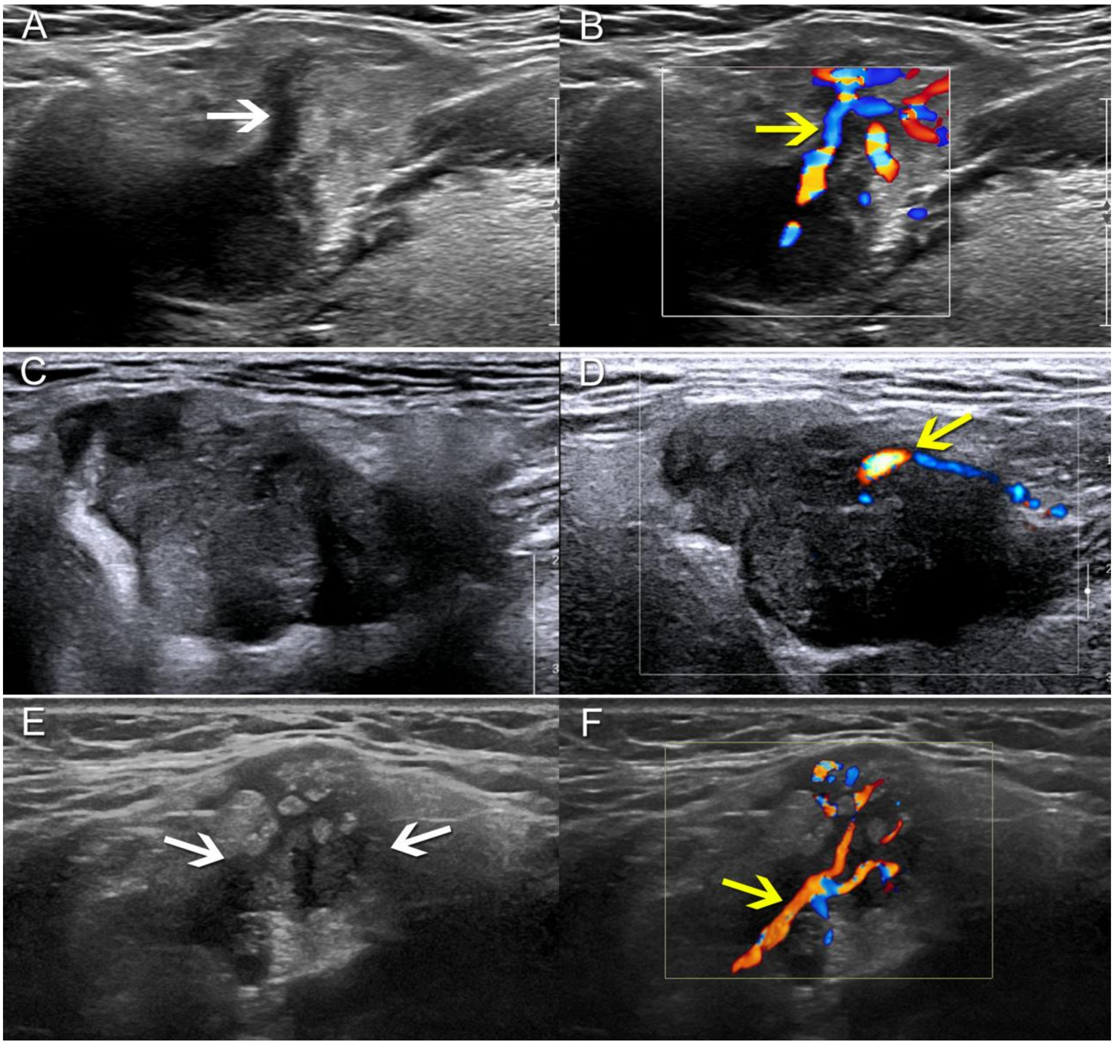

Supplementary Figure 1 US images of ACC in the salivary glands. **(A, B)** Case 1: a 35-year-old female with ACC in the right submandibular gland. (**A**) US image showed a 2.6 cm × 1.9 cm × 2.5 cm irregular nodule with a unclear border, absence of cystic areas, and presence of rat tail sign (white arrow). (**B**) CDFI showed a polar vessel (yellow arrow) as a dominant vessel without branches penetrating the nodule. **(C, D)** Case 2: a 66-year-old female with ACC in the right submandibular gland. (**C**) US image showed a 4.1 cm × 2.2 cm × 3.7 cm irregular nodule with a unclear border and absence of cystic areas. (**D**) CDFI showed a polar vessel (yellow arrow) as a dominant vessel without branches penetrating the nodule. **(E, F)** Case 3: a 57-year-old male with ACC in the right submandibular gland. (**E**) US image showed a 2.3 cm × 1.5 cm × 1.7 cm irregular nodule (white arrow) with a unclear border and absence of cystic areas. (**F**) CDFI showed a polar vessel (yellow arrow) as a dominant vessel with branches penetrating the nodule. Note: US, ultrasound; ACC, adenoid cystic carcinoma; CDFI, color Doppler flow imaging.

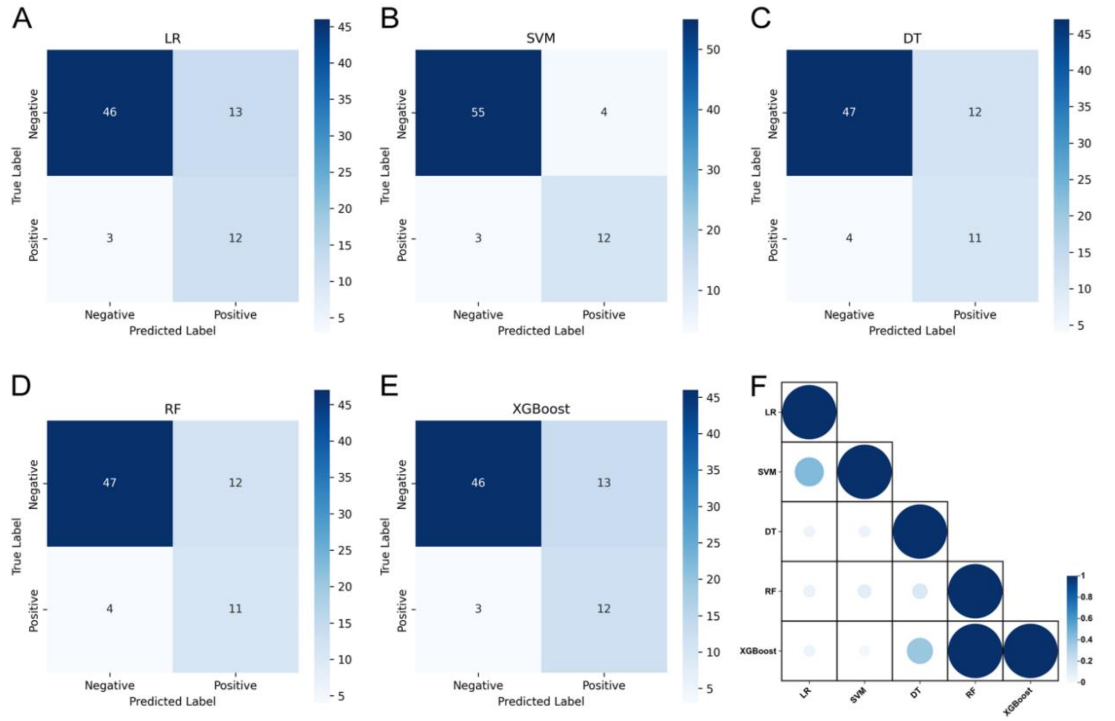

Supplementary Figure 2 Analysis of five ML models in the internal validation cohort: confusion matrices and AUC comparisons. **(A)** LR model. (**B**) SVM model. **(C)** DT model. **(D)** RF model. (**E**) XGBoost model. (**F**) *P* values for the pairwise comparison of AUC between different models. Note: ML, machine learning; LR, logistic regression; SVM, support vector machine; DT, decision tree; RF, random forest; XGBoost, extreme gradient boosting; AUC, area under curve.

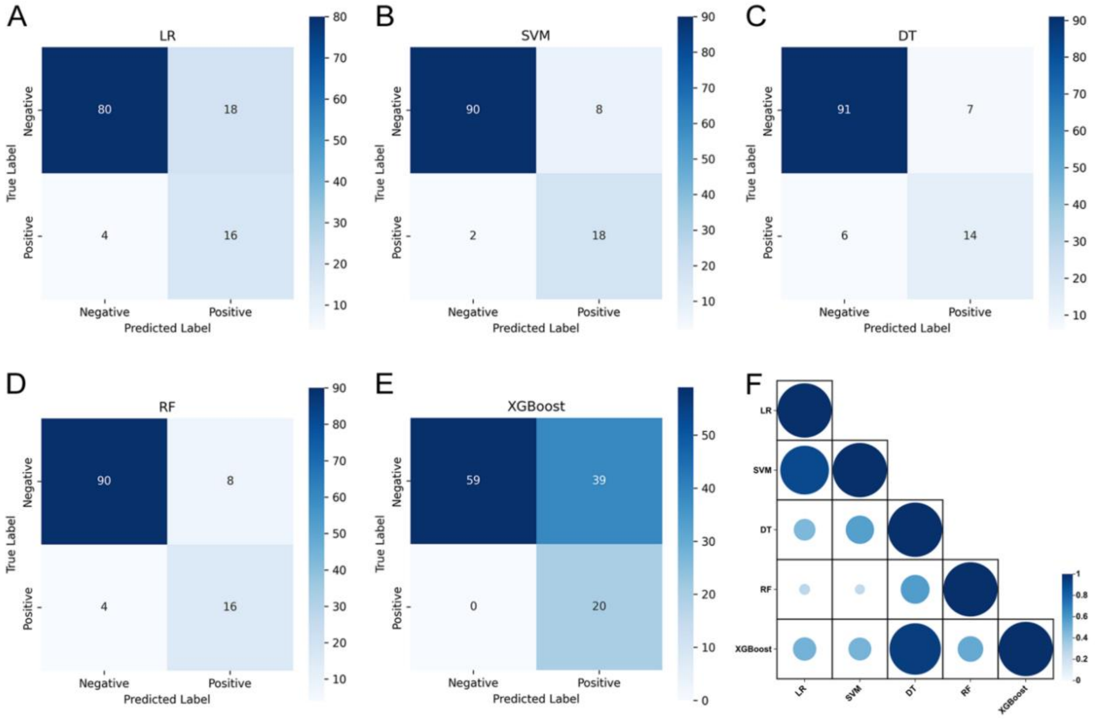

Supplementary Figure 3 Analysis of five ML models in the external validation cohort: confusion matrices and AUC comparisons. **(A)** LR model. (**B**) SVM model. **(C)** DT model. **(D)** RF model. (**E**) XGBoost model. (**F**) *P* values for the pairwise comparison of AUC between different models. Note: ML, machine learning; LR, logistic regression; SVM, support vector machine; DT, decision tree; RF, random forest; XGBoost, extreme gradient boosting; AUC, area under curve.

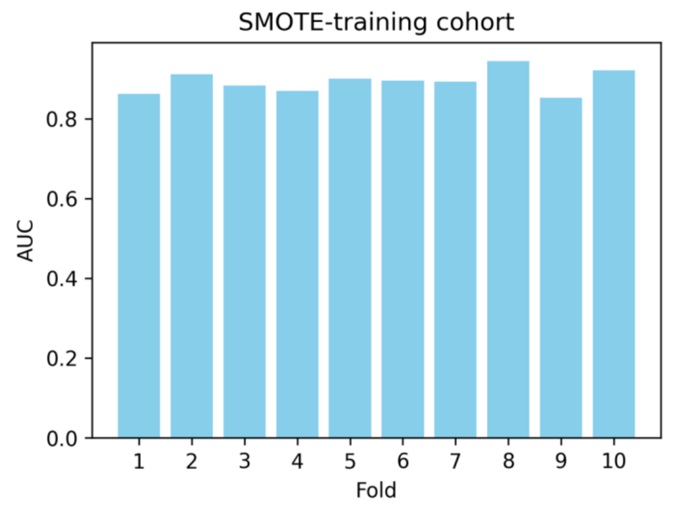

Supplementary Figure 4 The AUCs of 10-fold cross-validation for SVM model in the SMOTE-training cohort. Note: AUC, area under curve; SVM, support vector machine; SMOTE, synthetic minority over-sampling technique.
